# Some Insights into the Regulation of Cardiac Physiology and Pathology by the Hippo Pathway

**DOI:** 10.3390/biomedicines10030726

**Published:** 2022-03-21

**Authors:** Daniela Ramaccini, Gaia Pedriali, Mariasole Perrone, Esmaa Bouhamida, Lorenzo Modesti, Mariusz R. Wieckowski, Carlotta Giorgi, Paolo Pinton, Giampaolo Morciano

**Affiliations:** 1 Maria Cecilia Hospital, GVM Care & Research, 48033 Cotignola, Italy; dramaccini@gvmnet.it (D.R.); gpedriali@gvmnet.it (G.P.); ebouhamida@gvmnet.it (E.B.); 2 Laboratory for Technologies of Advanced Therapies (LTTA), Section of Experimental Medicine, Department of Medical Science, University of Ferrara, 44121 Ferrara, Italy; prrmsl@unife.it (M.P.); mdslnz@unife.it (L.M.); carlotta.giorgi@unife.it (C.G.); 3 Laboratory of Mitochondrial Biology and Metabolism, Nencki Institute of Experimental Biology, 02-093 Warsaw, Poland; m.wieckowski@nencki.edu.pl

**Keywords:** Hippo signaling, cardiac disease, cardiac physiology, YAP1, TAZ

## Abstract

The heart is one of the most fascinating organs in living beings. It beats up to 100,000 times a day throughout the lifespan, without resting. The heart undergoes profound anatomical, biochemical, and functional changes during life, from hypoxemic fetal stages to a completely differentiated four-chambered cardiac muscle. In the middle, many biological events occur after and intersect with each other to regulate development, organ size, and, in some cases, regeneration. Several studies have defined the essential roles of the Hippo pathway in heart physiology through the regulation of apoptosis, autophagy, cell proliferation, and differentiation. This molecular route is composed of multiple components, some of which were recently discovered, and is highly interconnected with multiple known prosurvival pathways. The Hippo cascade is evolutionarily conserved among species, and in addition to its regulatory roles, it is involved in disease by drastically changing the heart phenotype and its function when its components are mutated, absent, or constitutively activated. In this review, we report some insights into the regulation of cardiac physiology and pathology by the Hippo pathway.

## 1. Introduction

The Hippo pathway is a finely regulated and evolutionarily conserved molecular cascade involved in the control of tissue homeostasis and organ size that was first discovered through genetic screening studies in *Drosophila melanogaster* [1].

This signaling network is complex and involves several positive and negative modulators; all the proteins found in *Drosophila* have mammalian orthologs responsible for controlling cell survival, proliferation, and regeneration [2]. These usually refer to the canonical Hippo pathway, which is initiated by signals derived from the plasma membrane (PM) that are transmitted into the nucleus to activate the expression of several target genes [3].

### 1.1. Hippo Pathway Components

The first evidence regarding the importance of this pathway in cell fate derives from studies in fireflies in which the mutation of Hpo (Hippo) protein kinase led to apoptosis impairment and uncontrolled organ size [4]. Additionally, mutated Wts (Warts) kinase generated cell clones characterized by excessive growth and abnormal differentiation [5]. These two proteins are core components of the pathway in *Drosophila* and lead to a kinase-dependent cascade with the involvement of cofactors Salvador (Sav) and MOB-kinase activator-like 1 (Mats) and the Yorkie (Yki) transcriptional coactivator [6].

**Figure 1:**
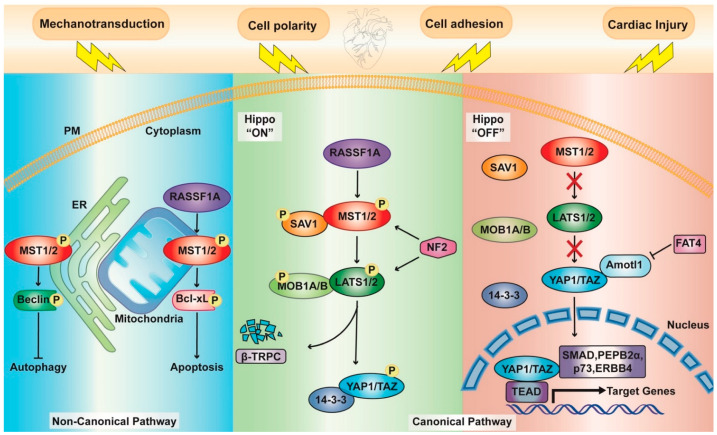
Schematic representation of the key components of the Hippo pathway. Canonical Pathway: In the “on” active state (**central panel**), upstream kinases (MST1/2, LATS1/2) together with their cofactors (SAV1 and MOB1A/B, respectively) are phosphorylated. Subsequential phosphorylation of YAP1/TAZ engages either 14-3-3 proteins for cytoplasmic retention or β-TrCP for ubiquitin-mediated protein degradation. In the “off” inactive state (**right panel**), upstream kinases are inactive; thus, YAP1/TAZ can translocate to the nucleus and bind with multiple transcription factors, including TEADs, SMAD, PEPB2α, P73, and ERBB4, thereby regulating several genes. The central components of the hippo pathway are regulated by several mechanisms and proteins, such as NF2, Amotl1, and FAT4. Noncanonical pathway (**left panel**): MST1 can directly phosphorylate either Beclin 1 at the ER, inhibiting autophagy in cardiomyocytes, or negatively regulating Bcl-xL at the mitochondria, enhancing cardiomyocyte apoptosis.

In mammals, homologous core components of the canonical Hippo pathway, namely, Hpo, Sav, Wts, and Mats, are the mammalian sterile 20-like protein kinase 1 and 2 (MST1/2), Salvador homolog 1 (SAV1), large tumor suppressor 1 and 2 (LATS1/2), and MOB kinase activator 1A and B (MOB1A and MOB1B), respectively; then, there are two Yorkie homologs: Yes Associated Protein 1 (YAP1) and WW Domain Containing Transcription Regulator 1 (TAZ) [7] (Figure 1).

Overall, the Hippo pathway exists in two different states: “on” and “off”. When the pathway is “on” active state, MST1/2 are phosphorylated; through a complex with their cofactor, SAV1 activates LATS1/2 kinases, which in turn interact with their regulatory proteins MOB1A and MOB1B. As a consequence, YAP1/TAZ become phosphorylated. This posttranslational modification prevents their translocation into the nucleus and promotes either cytosolic sequestration by binding 14-3-3 proteins or their degradation through β-transducin repeat-containing E3 ubiquitin-protein ligase (β-TrCP) [8].

In the “off” state, YAP1/TAZ are not phosphorylated, so they are able to bind a TEA domain transcription factor (TEAD) in the nucleus, leading to the transcription of downstream target genes [9]. As already reviewed in [6], YAP1/TAZ can also interact with other transcription factors, such as several Mothers Against Decapentaplegic Homolog 1 (SMAD) family members [10,11,12], p73 [13], ErbB-4 [14], and PEBP2α [15].

*Drosophila* mutagenesis studies have allowed the discovery of other upstream components in this pathway: two membrane-associated FERM domain (F for 4.1 protein, E for ezrin, R for radixin, and M for moesin) proteins Merlin (Mer) and Expanded (Ex) [16] and a WW domain protein Kibra, which interacts with Ex and physically interacts with Mer [17]. The homolog of Mer in mammals is neurofibromin 2 (NF2). This protein regulates Hippo signaling in response to the actin cytoskeleton state and directly interacts with LATS1/2 by recruiting them to the PM and promoting their phosphorylation [18].

### 1.2. Upstream Effectors

Hippo pathway activation has captured the attention of the scientific world, and to date, a plethora of effectors that intersect at different levels of the pathway have been defined. At least four groups of upstream pathways modulate the Hippo pathway and YAP1 localization (Figure 1). The first one is cell polarity and adhesion, which involves three major complexes, including Scribble, Crumbs, and Merlin [6,19,20]. The second is represented by the cadherin–catenin complex, in which it negatively controls nuclear YAP1, and the third group is orchestrated by soluble growth factors as extracellular signals [6]. Furthermore, Hippo signaling is regulated by mechanical signals and cell morphology, as demonstrated by the effect of F-actin fibers on the phosphorylation state of YAP1 [21] and the role of YAP1/TAZ as a sensor of matrix rigidity and cell shape [22]. More recently, several studies pinpoint the central role of Hippo pathway effectors as critical mediators of mechanical stress in the heart [23,24]. For instance, in 2020, Yamashiro et al. identified a matricellular glycoprotein, thrombospondin-1 (Thbs1), as an extracellular activator and integrin αvβ1-dependent of YAP1 in response to mechanical stress. By binding integrin αvβ1 and negatively regulating Ras-related GTPase Rap2, Thbs1 favors YAP1 translocation into the nucleus and consequent vascular remodeling [25]. In the same year, Arun A. and coworkers found increased levels of this protein in endothelial cells of patients infected by *Trypanosoma cruzi* and in those with several cardiovascular complications and heart damage [26,27]. In contrast, the group claimed that the upregulation of Thbs1 needed for early infection stages limits YAP1 localization into the nucleus. This apparent controversial finding may be an example of the tissue specificity that distinguishes this pathway. Indeed, the tissue-specific regulation of the activity of YAP1/TAZ, which are controlled in different ways depending on the tissue, must be noted [3,6].

### 1.3. A Noncanonical Point of View

Notably, YAP1 and TAZ might also be regulated by a noncanonical pathway in which the core kinase module does not directly target and modulate the cotranscription factors (Figure 1). Direct interactions between YAP1 and other proteins may support its restraint into the cytosol, similar to Ex-mediated sequestration [28]. Additionally, angiomotin (AMOT) family proteins are able to trap YAP1 and control the phosphorylation state of YAP1/TAZ by recruiting them to different compartments, such as tight junctions and the actin cytoskeleton [29]. Other cytoplasmic Ser/Thr kinases exist, and the most commonly described is MST4, which targets YAP1 to inhibit its import into the nucleus [30], TAK1 [31], MK5 [32], and AMPK [33]. Moreover, YAP1 and TAZ are modulated via ubiquitination–deubiquitination mechanisms (as reviewed in [34]) and by other posttranslational modifications, including methylation [35] and O-GlcNAcylation [36] (Figure 1).

Given the potential of the Hippo pathway in prosurvival mechanisms and the fact that the heart is unable to self-repair after injury, an understanding of its contribution in heart development, homeostasis, disease, and tissue regeneration may help in the identification of new molecular targets for cardio protection. For these reasons, we will summarize some aspects of cardiac development (in which apoptosis and autophagy work together in the recycling of the building blocks of macromolecules and the elimination of cells), cardiac disease, and the self-repair potential induced by the activity of the Hippo pathway.

## 2. The Hippo Pathway in Cardiac Physiology

### 2.1. Cardiac Development

Organ size regulation is one of the long-standing mysteries of biology, which is relatively constant and under the control of organ-intrinsic mechanisms and extrinsic physical and mechanical stress, in addition to circulating factors [37]. Throughout development, the mammalian heart size needs to be meticulously controlled to ensure proper blood circulation. Disorders in organ size (as part of the developmental process) are the most dramatic Hippo pathway phenotype alterations found in *Drosophila* [38] and many other organisms.

**Figure 2:**
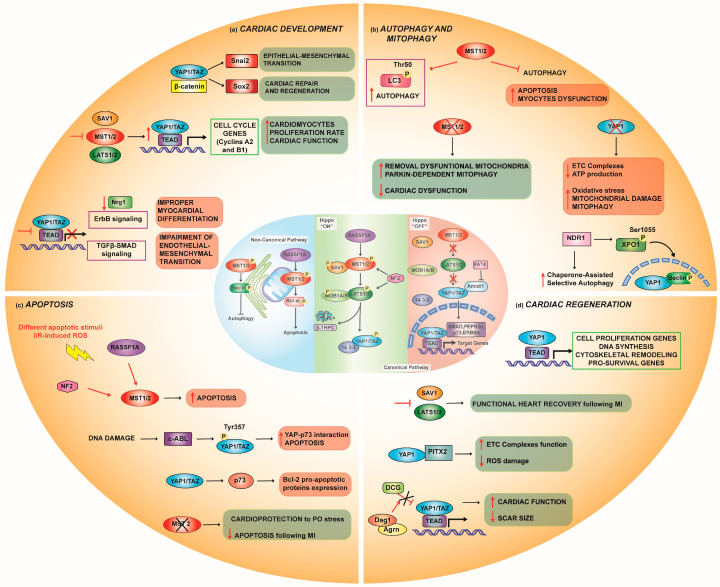
The Hippo pathway in cardiovascular physiology. (**a**) YAP1 expression is involved in cardiac development: the Wnt/β-catenin pathway synergically works with YAP1 to induce cardio genesis; nuclear YAP1 mediates cell cycle genes (cyclins A2 and B1), prompting cardiomyocyte proliferation; and YAP1 loss inhibits either ErbB or TGF-β-SMAD signaling with consequent improper myocardial development. (**b**) Hippo signaling controls autophagy and mitophagy: MST1/2 enhance autophagic flux through LC3 phosphorylation at Thr50; in contrast, under pathological conditions, MST1/2 both inhibits autophagy and boosts apoptosis. However, melatonin mediates autophagy activation through inhibiting MST1/2 phosphorylation and enhancing Parkin-dependent mitophagy. NDR1 kinase induces autophagy by YAP1 and Beclin1 translocation into the nucleus through XPO1 phosphorylation and modulates chaperone-assisted selective autophagy. Doxorubicin downregulates YAP1, leading to cardiac damage, which is the cause of activation of both autophagy and mitophagy. (**c**) Under pathological conditions, such as I/R, MST1/2 kinases are activated, leading to cardiomyocyte apoptosis; cytosolic YAP1 is stabilized by c-Abl phosphorylation at Tyr357, thus enhancing the YAP1-P73 interaction to activate apoptosis. (**d**) Inhibition of upstream kinases of the Hippo pathway, such as LATS1/2, promotes YAP1 nuclear translocation, thus targeting gene expression for cell proliferation and heart regeneration. YAP1 cardiac regenerative function is enhanced either by PITX2, which cooperatively reduces ROS damage, or by the Argn-Dag1 complex, which pulls apart DCG and improves cardiac function.

The Hippo pathway components are expressed in all three cardiac layers known as the myocardium, epicardium, and endocardium [38,39]. YAP1 expression plays a pivotal role in heart development; it is present at high concentrations in embryonic and neonatal animal hearts and disappears at approximately 12 weeks of age [40]. It triggers cell proliferation in vitro by regulating cell cycle genes (mainly cyclins A2 and B1) and requires TEAD binding (Figure 2a). Notably, forced induction of YAP1 is able to activate the genetic program involving proliferation in cells that have exited the cell cycle (i.e., adult differentiated cardiomyocytes) [40]. Heallen and colleagues first demonstrated the important roles of the Hippo pathway in controlling heart size in animal models [41], taking advantage of several genetically modified mice. To induce the hyperactivation of YAP1, they used the Nkx2.5-Cre-mediated loss of SAV1, MST1/2, and LATS2 Hippo components. This genetic background produced a thickening of compact ventricular myocardium in favor of cardiomegaly and trabecular expansion in embryonic mouse hearts [41]. Despite the drastic changes in the myocardium morphology, cardiomyocyte size was unaffected, while their proliferation rate was significantly upregulated [40,41]. In this setting, natriuretic peptide A (Nppa) expression, an established marker of trabecular myocardium, was extensively reduced, which linked the upregulated cardiomyocyte proliferation with an altered differentiation state [40].

In agreement, mouse hearts with *Tnnt2*-Cre-mediated embryonic deletion of YAP1 exhibited embryonic lethal cardiac hypoplasia and severely thinned myocardial layers with reduced cardiomyocyte proliferation [40,42]. Additionally, deletion of YAP1 in the embryonic heart leads to lethality at E10.5 [43]; when depleted postnatally, increased myocardial fibrosis, cardiomyocyte apoptosis, and decreased cardiomyocyte proliferation occur [42,44].

By using a different approach, or the endocardial-specific loss of YAP1 and TAZ (Nfatc1-*IRES*-Cre), Artap and coworkers found improper myocardial formation, the first cause of postnatal lethality in these transgenic animals. YAP1/TAZ absence in the myocardium resulted in a significant downregulation of neuregulin 1 (Nrg1) expression, which is an important endocardial factor that expresses the cell surface ligand ephrin and orchestrates myocardium differentiation/phenotype [45]. To demonstrate the specificity of the effects on Nrg1 mediated by the presence/absence of YAP1, it has been shown how YAP1 can bind to the promoter sequences of Nrg1 and activate ErbB signaling, which is critical for the function of myocardium development [45] (Figure 2a).

#### 2.1.1. Interaction with Other Pathways in Cardiac Development

In many aspects of cardiac development, the Hippo pathway exerts its functions by taking advantage of crosstalk with other routes, such as the Wnt/β-catenin and insulin growth factor (IGF) pathways, transforming growth factor (TGF)-β-SMAD and Ras association family member 1 isoform A (RASSF1A) signaling.

##### Wnt/β-Catenin Pathway 

Similar to the Hippo pathway, the Wnt pathway is crucial in cardio genesis, especially in cardiac overgrowth. In a mouse model of SAV1 KO, negative regulation of Wnt/β-catenin by the Hippo pathway has been found in embryonic hearts. YAP1 and β-catenin physically interact with the *Snai2* (regulating epithelial–mesenchymal transition, EMT) and *Sox2* (regulating cardiac repair and regeneration) genes and affect their transcription [41] (Figure 2a). In support of these findings, many groups of researchers have achieved similar results by analyzing the gain of function of activated YAP1 in neonatal and perinatal hearts. Transgenic mice overexpressing the constitutively active form of YAP1 had sustained levels of β-catenin and were positive for the enhanced fluorescence of phospho-histone H3 (pHH3) and cardiac troponin, an index of increased myocyte proliferation during development compared to wild-type (WT) littermates. This phenotype was accompanied by and sufficient to stimulate an overgrowth of the heart [42]. Additionally, Monroe et al. reported that completely differentiated cardiomyocyte-specific overexpression of YAP1 restored features of fetal-like cell states by increasing access to embryonic cardiac enhancers in the adult heart [46]. Influencing cell cycle gene expression, cytoskeletal remodeling, and Wnt signaling, YAP1 led to enhanced proliferation, resulting in myocardium thickening and improved cardiac function [46]. Notably, when the expression of YAP1 is driven by the β-MHC promoter, adult heart size is normalized due to reduced cardiomyocyte size, although the cell numbers are higher than those of normal controls [42]. Under this specific YAP1 activation condition, the reason for the interplay between cell number and cell size to maintain a set heart size is obscure and fascinating.

##### IGF Pathway

Studies on cardiomyocyte mice overexpressing YAP1 showed enhanced IGF signaling through the transcription of IGF1 and IGF binding protein (bp2/3) genes [43] and an increase in Akt phosphorylation. In turn, the inhibition of the IGF pathway and the loss of β-catenin stabilization have a negative effect on YAP1-dependent cardiomyocyte proliferation. This information further validates the importance of YAP1 in the activation of the IGF and β-catenin pathways.

##### TGF-β-SMAD Pathway

The correlation between the Hippo pathway mediator YAP1 and the TGF-β-SMAD-2/3/4 pathway, which is critical for the proper development of the atrioventricular cushion, has been documented. YAP1 loss of function has been found to alter TGF-β-SMAD signaling in endothelial cells and thus impair endothelial mesenchymal transition (EMT) genes, including *Twist1*, *Snail1*, and *Slug*, indicating the important roles of the Hippo pathway in the regulation of endocardial/endothelial cells in heart development [47] (Figure 2a).

##### RASSF1A Signaling

RASSF1A is a member of the Hippo pathway and is ubiquitously expressed and detected in cardiac tissue [48,49]. RASSF1A has an inhibitory effect on cardiac growth and cell survival through its intersection with the Hippo pathway. This happens through various protein–protein bindings at multiple levels: (i) by allowing increased interaction between MST2 and LATS1 kinases; (ii) by directly binding MST2 [50]; and (iii) by interacting with a SAV1 member [51]. This, with the final goal of potentiating YAP1 translocation into the nucleus and its interaction with p73 and its stabilization to increase apoptosis induction [52] (Figure 2c).

Moreover, RASSF1A interacts with PM calcium ATPase (PMCA) 4b, mediating the alleviation of extracellular signal-regulated kinase 1/2 (ERK), suggesting a further contribution of RASSF1A in modulating cardiac growth in neonatal rat cardiomyocytes [53].

Development not only involves cardiomyocyte proliferation during fetal stages and cell hypertrophy in the postnatal heart but other events occur to maintain the correct balance between cell life and cell death, such as autophagy and apoptosis. Both are also involved either in transitory stressful conditions or in cardiac pathologies where autophagy may be activated as a prosurvival mechanism of defense against injury to limit excessive apoptosis and the loss of cardiac function. As the Hippo pathway is reported to modulate both pathways, knowledge of its contributions is essential to understand what becomes deranged in disease.

### 2.2. Autophagy

Autophagy is a self-digestion process that occurs in response to cellular stresses, including hypoxia, starvation, infection, and enhanced oxidative stress [54]. Autophagy targets cytoplasmic components to lysosomes for degradation and for the recycling of building blocks in response to cell stress [55,56]. During this process, harmful cytosol-localized components, including invading pathogens, damaged organelles, and protein aggregates, can selectively be removed to ensure a healthy cell population [55,57].

However, autophagy represents a double-edged sword in the heart. Although it is primarily a prosurvival mechanism, excessive autophagy can destroy key cellular components or autophagic machinery itself, resulting in cell death. Indeed, part of the literature considers it to be at the crossroads between cell survival and death [58].

Recently, the Hippo signaling pathway has been recognized as a process controlling autophagy. A primary role comes from the observations that both MST1 and MST2 contribute to autophagy regulation under resting conditions through the phosphorylation of LC3 at threonine (Thr) 50 [59] (Figure 2b). MST1/2-dependent autophagic flux is conserved among taxa and is needed for autophagosome–lysosome fusion to ensure intracellular bacterial clearance. Accordingly, their loss is accompanied by severe defects in autophagy [59]. Nonetheless, in the heart and under pathological conditions, the role of MST1 as an inhibitory mechanism toward autophagy, leading to an accumulation of p62 and protein aggregates in cardiomyocytes, has been documented [60]. Here, MST1 alters pro-autophagic Atg14L-Beclin-Vps34 complex formation and promotes, in a YAP1-independent manner, the phosphorylation of Beclin 1 in its Bcl-2 homology 3 (BH3) domain at Thr108. This phosphorylation cascade can enhance the interaction between Beclin 1 and the antiapoptotic proteins Bcl-2 and Bcl-2-like protein 1 (Bcl-XL), thus favoring apoptosis and myocyte dysfunction [60] (Figure 1). These controversial results may be due to the investigation of two quite different processes: xenophagy and autophagy.

Additionally, a couple of protein kinases belonging to the family of LATS1/2, downstream of MST1/2, and named nuclear Dbf2-related (NDR1/2) kinases [61], also play a major role in the response to cellular stress-inducing autophagy [62] (Figure 2b). It was first reported that NDR1 phosphorylates YAP1 at the same localization site as LAST1/2 [62,63], thus modulating its cytosolic-nuclear shuttling inside cells. NDR1 is a protein that contributes to nutrient starvation-induced autophagy by phosphorylating exportin 1 (XPO1) at serine (Ser) 1055, a nuclear receptor involved in the translocation of proteins from the nucleus to the cytoplasm. In this case, NDR1-mediated phosphorylation allows the passage of autophagic regulators, such as Beclin 1 and YAP1, across the nucleus [64]. Furthermore, it has been demonstrated that NDR1 modulates chaperone-assisted selective autophagy (CASA), a molecular pathway in which mechanical forces induce a form of autophagy that targets the filamin protein. It is orchestrated by the CASA complex, which is composed of mutual interactions of HSP70, Synaptopodin 2, Heat Shock Protein Family B (Small) Member 8 (HspB8), BAG Cochaperone 3 (BAG3), and the ubiquitin ligase CHIP [65] (Figure 2b).

Additionally, melatonin is known to regulate the autophagic process in different ways [66]. One of them acts through the MST1/SIRT3 signaling pathway; indeed, melatonin is able to prevent MST1 phosphorylation and elevate the levels of Beclin 1, LC3-II, and ATG5 [67]. These molecular pathways appear to be interconnected when melatonin failed to induce cardioprotective effects in transgenic MST1 KO mice [67]. Melatonin induces autophagy in the heart by suppressing the MST1-mediated cascade [67].

#### Mitophagy

Mitophagy is an autophagic response that specifically targets damaged mitochondria; it plays a pivotal role in mitochondrial quality control and protects the heart from pathological stress through multiple pathways [68,69,70]. Among those that are dependent on the Hippo pathway, Parkin-dependent mitophagy activation upon MST1 suppression [68] emerges as a crucial protective step in some types of CMs to remove dysfunctional and harmful mitochondria [71] (Figure 2b). This finding has been further confirmed recently by Shang et al. in a lipopolysaccharide (LPS)-induced septic cardiomyopathy model in which MST1 deletion resulted in an upregulation of mitophagy through Parkin, thereby protecting mitochondria from LPS-dependent alterations and cell death [72].

Additional proof regarding the beneficial effects of YAP1 activation in the heart comes from the analysis of mitophagy when YAP1 is depleted. In the absence of YAP1, excessive mitophagy led to a reduction in the expression of complexes I–IV of the electron transport chain (ETC) and contributed to insufficient ATP generation [73]. Furthermore, cellular oxidative stress and the suppression of several mitochondrial genes prompted mitochondrial-mediated apoptosis [74].

### 2.3. Apoptosis

Apoptosis is a fundamental process with the aim of removing useless or damaged cells during development and preserving organism homeostasis [75,76]. The Hippo pathway and signaling components are well recognized as fundamental regulators of apoptosis [7,77,78]. In *Drosophila*, the “on” state of the Hippo pathway is required for inactivating the transcriptional coactivator Yki; a failure in doing so results in uncontrolled tissue overgrowth for defective apoptosis [7,77,79]. In contrast, when Yki is activated, it promotes growth by stimulating cell proliferation and inhibiting apoptosis. The mechanism through which it achieves this was partially elucidated by Thompson and Cohen in 2006. They showed that Yki can promote the expression of *bantam*, a microRNA known to be a regulator of both proliferation and apoptosis [80]. When *bantam* is overexpressed, it rescues cells from apoptosis, thus mimicking Yki activation, and can promote growth in cells expressing decreased levels of Yki. Conversely, deletion of *bantam* blocks Yki-driven overgrowth [81,82]. Hence, these results highlighted the role of the Hippo pathway in the regulation of the expression of *bantam* to control tissue growth in *Drosophila*.

In the same organism, apoptosis is also mediated by the Yki/Scalloped complex, which promotes the transcription of the inhibitor of apoptosis (IAP) Diap-1 [83,84], a member of the IAP family and known to be an important modulator of apoptosis in developing *Drosophila* tissues [85,86]. Specifically, Diap-1 inhibits the *Drosophila* caspases Dronc and DrICE. Therefore, the enhanced Yki-dependent transcription of Diap-1 functions as a powerful anti-apoptotic mechanism that promotes tissue overgrowth.

More focused on the cardiac field and mammals, the role of the Hippo components in apoptosis has mainly emerged when analyzing pathologic phenotypes. For example, MST1/2 kinases are known to be activated by various apoptotic stimuli even before their role in the Hippo pathway was elucidated [87]. Among them, oxidative stress is considered one of the most relevant insults that trigger MST1/2 activation. Ischemia/reperfusion (I/R) is recognized as one of the most common injuries to human hearts that leads to cardiomyocyte death predominantly following ROS overproduction [88]. Accordingly, the potential regulation of MST1/2 by I/R-induced ROS and the role of MST1/2 in myocardium injury have been largely investigated [49,89,90]. Zi and coworkers revealed that MST2 KO results in a protective effect in the heart under pressure overload (PO) stress. MST2 KO mice also displayed less apoptosis and fibrosis after transverse aortic constriction (TAC) stimulation [91]. Similarly, inhibition of MST1 prevented cardiomyocyte apoptosis and protected against cardiac dysfunction following myocardial infarction (MI) [90]. In line with this finding, overexpression of MST1 led to harmful excessive apoptosis [89]. Furthermore, overexpression of dominant-negative MST1 in mice leads to reduced infarct size after MI and hence decreased cardiomyocyte apoptosis [92].

The pro-apoptotic function of MST1/2 is also stimulated by RASSF1A through a noncanonical mechanism, resulting in the inhibition of Bcl-XL [93] (Figure 1). Interestingly, in cardiomyocytes, it has been reported that increased RASSF1A expression activates MST1 to inhibit cell growth and promote apoptosis. Nevertheless, in cardiac fibroblasts, RASSF1A prevents cell proliferation while inducing apoptosis through MST1, thus highlighting an opposite effect between both cell types [94] (Figure 1 and Figure 2c).

Beyond the role of RASSF1A in MST1 activation, it has also been reported that neurofibromin 2 (NF2) is responsible for MST1 activation in cardiomyocytes. Indeed, the activation of NF2 by oxidative stress induces complex formation between MST1 and LATS2, which promotes MST1 activation during I/R. NF2 conditional KO mice showed significantly smaller infarcts with diminished cardiomyocyte apoptosis and improved heart function after I/R [95]. This evidence suggests that endogenous MST1 is an important mediator of apoptosis between physiology and pathology.

**Figure 3:**
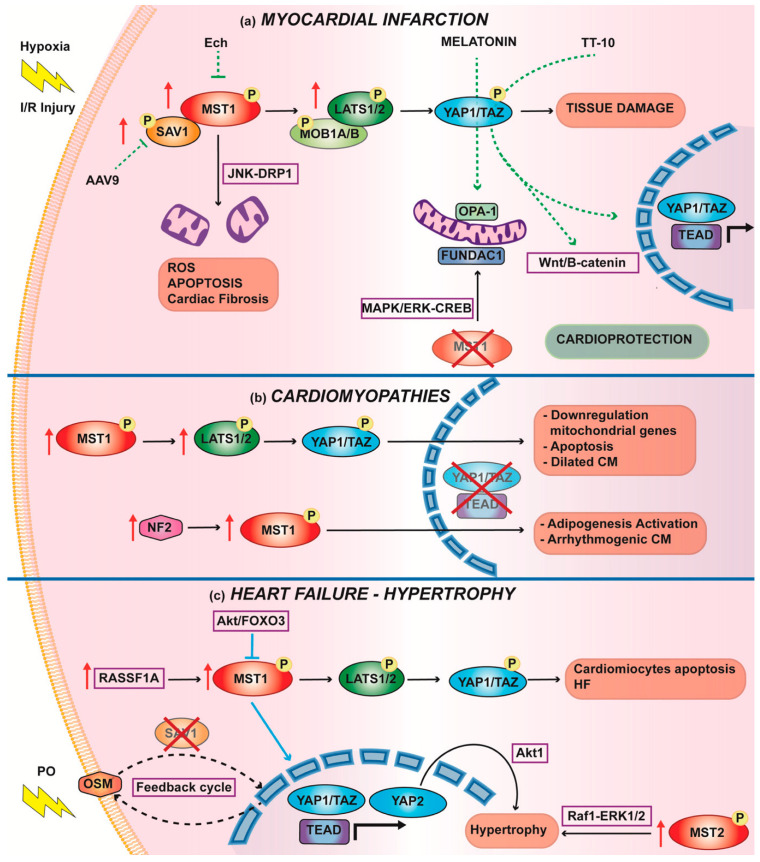
The Hippo pathway in cardiac disease. (**a**) In myocardial infarction, the Hippo pathway is upregulated. Overexpression of MST1 activates mitochondrial fragmentation via JNK-DRP1, enhancing cardiomyocyte apoptosis and cardiac fibrosis. In contrast, its deletion activates mitophagy via MAPK/ERK-CREB. Green arrow dashed lines indicate cardioprotective treatments to turn off the Hippo pathway. (**b**) The activation of upstream kinases leads to the downregulation of mitochondrial genes and the upregulation of cardiomyocyte apoptosis, ultimately leading to lethal Dilated CM. Arrhythmogenic CM is characterized by adipogenesis activation, which is secondary to overexpression of NF2 and upregulation of upstream kinases. (**c**) Heart failure exhibits overexpression of RASSF1A, enhancing phosphorylation of MST1 and consequently YAP1 cytosolic retention. Upregulation of the Akt/FOXO3 pathway inhibits MST1 activation; thus, YAP1 translocates into the nucleus, leading to cardiac hypertrophy. Additionally, nuclear YAP2 induces cell proliferation and hypertrophy via Akt1 signaling. MST2 overexpression also induces cardiac hypertrophy via the Raf1-ERK1/2 pathway but not through Hippo signaling. SAV1 deletion shows a positive feedback cycle of YAP1/TEAD-OSM, which exacerbates cardiac injury after PO.

Nonetheless, increasing evidence suggests a dual role for YAP1 in mammals. Indeed, it can both induce and suppress apoptosis. On the one hand, the pro-apoptotic activity of YAP1 is ascribed to its ability to activate p73 (a member of the p53 family) [96] and induce the expression of pro-apoptotic components of the Bcl-2 family [52]. Interestingly, Levy and coworkers reported that in response to DNA damage, the tyrosine kinase c-Abl directly phosphorylates YAP1 at Tyr357, thus stabilizing it. This event increases the nuclear interaction of YAP1-p73 to activate the apoptotic program [97] (Figure 3c).

In addition, YAP1 and TAZ can also protect the cell against anoikis, and the inactivation of YAP1 mediated by LATS1/2 helps drive this type of cell death [98,99]. It is now becoming increasingly clear that the Hippo pathway has an important, evolutionarily conserved role in the regulation of the apoptotic response.

## 3. The Hippo Pathway in Cardiac Diseases

As many cardiac diseases are leading causes of death in the world and the heart is an organ that is unable to self-repair after insults, it is crucial to establish adjuvant cardioprotective therapies in addition to the current clinical practice. Being in the middle of many prosurvival mechanisms and modulating both autophagy and apoptosis, the Hippo pathway is considered an important field of research for cardio protection. In the following sections, we considered the three most important phenotypes of cardiac diseases: myocardial infarction (MI), cardiomyopathy (CM), and cardiac hypertrophy (CH), which are considered risk factors for heart failure (HF) (Figure 3).

### 3.1. Myocardial Infarction

MI is a disease generated by an impairment of blood flow (including oxygen supply) due to an obstruction of one or multiple coronary arteries, which leads to cardiomyocyte damage and death. The current gold-standard therapy for MI is mechanical reperfusion; paradoxically, this leads to further tissue damage called ischemia/reperfusion injury (IRI). Several processes concur with this phenomenon and include ROS overproduction, inflammation, and mitochondrial dysfunction [100].

Mimicking MI both in vitro and in vivo led to the identification of a lower amount of nuclear YAP1 (Figure 3a). Although an increase in MST1/2 [89,90], SAV1 [101], and LATS1/2 [102] transcripts has been found after hypoxia, IRI triggered a drop in the levels of nonphosphorylated MST/LATS proteins [103]. In the same context, apoptotic stimuli, such as caspase-dependent cleavage, activate MST1 and further control myocyte death through apoptosis [89]. Mechanistically, one molecular pathway in which MST1 is involved is enhanced mitochondrial fission via the JNK-Drp1 pathway [104]. Increased mitochondrial fission was accompanied by the inhibition of mitophagy due to a concomitant decrease in FUNDC1 expression [105]. Accordingly, the in vivo cardiac-specific overexpression of MST1 dominant-negative (MST1 K59R) revealed cardio protection with reduced infarct size [89,90]. Additionally, MST1-knockout cardiomyocytes were protected from injury by the effect of MST1 on sustaining mitochondrial homeostasis, ROS production, mitochondrial membrane potential, and mPTP opening. This genotype preserved the activation of FUNDC1 via the MAPK/ERK-CREB pathway with a protective role in maintaining mitochondrial homeostasis through mitophagy [105].

From these findings, MST1 (and with him the Hippo pathway) acts at multiple levels of mitochondrial function following I/R, impairing several mechanisms of quality control.

#### Therapies to Increase Nuclear YAP1 to Counteract IRI

In 2019, Khan K. and coworkers investigated the effects of the constitutively active form of YAP1 in a model of human ventricular cardiomyocytes subjected to hypoxia and reoxygenation. YAP1-induced expression first reduced apoptosis, prevented hypertrophy, and attenuated ROS generation during reperfusion [103]. Second, it contributed to the activation of Wnt signaling, strengthening the beneficial effects. One possible therapy for the efficient induction of YAP1 nuclear expression may be AAV9-based gene therapy, which has already been reported to counteract the activation of the Hippo pathway [44,101,106] (Figure 3a).

A second strategy is suggested by some cardioprotective compounds that are known to act synergistically with the Hippo pathway. For example, Echinatin (Ech), a component of the traditional herb Glycyrrhiza, inhibits MST1, LATS1, and YAP1 phosphorylation both in vitro and in vivo, favoring YAP1 translocation into the nucleus and its activation [102]. Melatonin has been deeply studied for its protective role after I/R, and its activity has been linked to the activation of OPA1-related mitochondrial fusion with the involvement of the Hippo pathway, although the precise mechanism by which YAP1 governs OPA1 expression is not yet clear [107]. Through chemical screening, a promising drug named TT-10, acting on YAP1-TEAD1 activity and the Wnt/B-catenin signaling pathway, improved the cardiac function of mice after MI, acting on cell proliferation [108] (Figure 3a). Subsequent studies on human induced pluripotent stem cell (hiPSC)-derived cardiomyocytes (hiPSCMs) confirmed the effects of this drug on the cell cycle and division without side effects on functional or structural genes of these cells, enhancing its potential role in clinical treatment [109].

Although numerous studies have reported beneficial effects of the inactivation of the Hippo cascade in cardiomyocytes following MI, it should be reported in a very recent work in which YAP1 and TAZ assumed an opposite function in cardiac fibroblasts, inflammatory responses, and profibrotic pathways that worsen the phenotype of the remodeled heart were promoted [110].

### 3.2. Cardiomyopathies

CMs are a heterogeneous group of heart diseases that affect structural and functional myocardial function. A variety of phenotypes and etiologies are involved; therefore, a specific classification is constantly evolving. Signs and symptoms mostly overlap, and they possibly share molecular mechanisms and gene mutations [111]. Among them, dilated (DCM) CM is an idiopathic CM that presents as HF, which is secondary to left ventricular dilatation and systolic dysfunction.

Several studies have suggested the activation of the Hippo pathway and the consequent YAP1 inhibition before DCM onset. Transgenic mice with cardiac-specific overexpression of MST1 showed an increase in cardiomyocyte apoptosis, which led to DCM. However, these cardiac myocytes were smaller, and the mice died within 2 weeks [89]. With the same phenotype, a mouse model of conditional cardiac-specific deletion of YAP1 exhibited a high level of apoptosis. These mice died within 20 weeks due to lethal DCM [44]. Accordingly, a murine model of LATS2 cardiac overexpression also showed DCM [95].

Growing evidence indicates a connection between the Hippo pathway and mitochondrial dysfunction related to DCM [112]. Loss of TEAD1 leads to lethal acute-onset DCM [113,114]. Using a tamoxifen-inducible adult CM-specific TEAD1 mouse model, the authors first demonstrated that TEAD1 loss impaired sarcoplasmic reticulum calcium homeostasis due to loss of SERCA2a activity, which contributed to the impairment of excitation–contraction coupling. Moreover, in their last work, they highlighted how the loss of TEAD1 downregulated mitochondrial genes, such as mitochondrial ETC, and genes encoding for enzymes in fatty acid oxidation. Accordingly, another independent study using a transgenic MST1 mouse model exhibited the downregulation of several nuclear DNA (nDNA)-encoded mitochondrial gene sets [115]. Mechanistically, a reduced physical interaction between YAP1-TEAD1 led to a repression of activation of nuclear transcription factors of mitochondrial genes [74] (Figure 3b).

Some data suggest a role for Hippo signaling in the pathogenesis of arrhythmogenic CM. This is characterized by right ventricular cardiomyocyte replacement with fibro-adipocytes and is, therefore, also known as arrhythmogenic right ventricular cardiomyopathy. Mutations in several genes encoding intercalated disk proteins have been detected in human patients with arrhythmogenic CM [116]. Chen et al. observed overactivation of NF2 both in human samples and mouse models. NF2 is an upstream molecule that triggers phosphorylation of the Hippo kinases (MST1/2, LATS1/2, and YAP1), further suppressing YAP1-TEAD1 transcription gene activation and enhancing adipogenesis [117] (Figure 3b).

### 3.3. Hypertrophy and Heart Failure

Hypertension, cardiac hypertrophy, diabetes, MI, and CMs are all risk factors for HF. As we found that the Hippo pathway was more or less involved in all these pathological states, it is reasonable to think that it continues to be involved in HF. Accumulating evidence has shown that Hippo pathway activation occurs during HF by inducing cardiac myocyte apoptosis and maladaptive phenotypes.

Del Re et al. showed that in the stressed heart, MST1 is activated endogenously when phosphorylated by RASSF1A, which results in significant upregulation [94]. In this scenario, RASSF1A, as an upstream and interacting member of MST1, exhibits deleterious functions for the heart, and its downregulation improved cardiac workload (Figure 3c).

One of the recognized methods to induce cardiac hypertrophy and HF in animal models is TAC [118]. LATS2 is an endogenous regulator of cardiac hypertrophy during TAC [95]; in detail, Matsui et al. suggested that in response to PO, there was an upregulation of endogenous LATS2, which increased cardiomyocyte MST1-mediated apoptosis and inhibited adaptive cardiac hypertrophy [95]. Accordingly, the expression of a dominant-negative LATS2 inhibited cardiac myocyte death triggered by TAC. Even MST2 has been seen to induce cardiac hypertrophy after PO when it is overexpressed. However, its effect seems to be exerted not through YAP1 but upon activation of Raf1-ERK1/2 [91].

Since upstream kinases are activated during HF and promote cardiomyocyte apoptosis and reduce cell proliferation, it has been questioned whether upregulation of the nuclear effector of the Hippo pathway, YAP1, could also have a cardioprotective effect in this field. Several studies have tried to elucidate its role in cardiac hypertrophy and HF; however, its function seems to be complex and context-dependent. Heterozygous cardiac-specific YAP1-KO mice exhibit increased cell apoptosis and fibrosis; however, adaptive cardiac hypertrophy is attenuated after MI [44] and acute PO [119]. Wang et al. showed that in human heart samples from patients with hypertrophic CM and mice following TAC, phosphorylation at Ser127 of YAP1 is reduced. Hence, increased nuclear expression of YAP1 induces transcription of hypertrophic genes [120]. Moreover, they also found a concomitant reduction in the levels of MST1 by the Akt/FOXO3 pathway. In contrast, Lin et al. found that adult mice with cardiac-specific activation of YAP1 had a cardioprotective role in the long term after MI. In this context, YAP1 seems to exert its protective role by promoting cardiomyocyte proliferation rather than inducing compensatory cardiac hypertrophy [106].

Recently, Song et al. highlighted the role of YAP2 in myocardial hypertrophy. They confirmed in vitro, in vivo, and in human samples that YAP2 overexpression induces cardiomyocyte hypertrophy. Mechanistically, they showed that YAP2 exerted its function through activation of Akt, thus promoting cell proliferation and hypertrophy [121]. These results indicated that either the inhibition of the Hippo pathway or upregulation of YAP1 led to cardiomyocyte proliferation, triggering cardiac hypertrophy. However, a new work by Sadoshima’s group underlines that prolonged activation of either YAP1 or the chronic suppression of the upstream pathway is detrimental in the presence of long-term PO [122]. The cardiac-specific SAV1-KO mouse model under 12 weeks TAC exhibited a positive feedback cycle of YAP1/TEAD1- OSM (oncostatin M), which exacerbated cardiac injury (Figure 3c). Certain levels of YAP1 are necessary to induce adaptive cardiac hypertrophy after PO. However, when it is long-term overexpressed, it becomes detrimental for the heart and leads to HF [47].

The extracellular matrix (ECM) provides structural support to tissues, and in the heart, it plays a pivotal role in its physiology; in the development of HF, the ECM is deranged and remodeled, deviating substantially from its original complexity. Major phenotypes are represented by cardiac fibrosis, which mainly alters ECM structure. As mentioned in the introduction, ECM is a place where many stimuli affecting the Hippo pathway reside. A seminal paper by Perestrelo et al. explained how mechanical stress caused by MI on ECM activates and sustains mechanosensitive YAP1 in cardiac fibroblasts, which together with TGF-β1, induces profibrotic cardiac remodeling in a positive loop [123]. It should be noted that pericytes ameliorate cardiac function and enhance cardiac repair after myocardial ischemia via attenuation of cardiac remodeling, alleviation of inflammatory responses, and induction of angiogenesis; however, after chronic ischemia and in failing hearts, they display altered mechanotransduction properties characterized by reduced expression and translocation to the nucleus of YAP1 and the consequently decreased transcription of angiogenic factors, such as dimethylarginine dimethylaminohydrolase 1 (DDAH1), connective tissue growth factor (CTGF), and cysteine-rich angiogenic inducer 61 (CYR61) [124].

In the context of pathological ECM remodeling associated with cardiac fibroblast differentiation into myofibroblasts, the YAP1/TAZ pathway overlaps with other molecular routes, such as those indicated by TGF-β and WNT proteins [11,125,126,127]. Although crosstalk among these pathways in cardiac physiology has been reported, the interpathwaycommunication during cardiac fibrosis evolution has only recently been highlighted. Recent work by Mia et al. demonstrated that YAP1 mediates fibroblast activation by acting downstream of both WNT and TGF-β [110], modulating fibroblast proliferation, and polarization of macrophages to become proinflammatory in the cardiac zone of interest. Another study highlighted an upregulation of endogenous YAP1 in cardiac fibroblasts from human heart samples with HF, which was accompanied by a downregulation of LAST1 [128]. Accordingly, conditional deletion of LAST1/2 results in myofibroblast transformation [129].

## 4. Cardiac Regeneration

While lower vertebrates retain a remarkable capacity for cardiac regeneration throughout life [130,131,132], the mammalian heart maintains its regenerative capability only during early life and loses this peculiarity postnatally [133,134,135]. Therefore, as we have seen previously, loss of cardiomyocytes after heart injury leads to pathological consequences, eventually leading to sudden death [136].

In this context, the Hippo cascade has been recently found to be of primary interest in supporting cardiac regeneration. Indeed, either the repression of Hippo kinase modules or YAP1 activation significantly provides an attractive therapeutic target for promoting cardiomyocyte renewal and cardiac regeneration in the adult heart [78,137].

Increasing evidence supports the idea that the Hippo signaling pathway is a critical barrier to cardiac regeneration. Interestingly, YAP1 is not only essential for cardiomyocyte proliferation during mouse embryonic cardio genesis [40,43] but is also crucial for adult cardiomyocyte homeostasis [44]. As seen in cardiac development, if analyzed in the adult heart, the activation of YAP1 may represent an effective strategy for promoting heart regeneration after injury. Indeed, YAP1 induces the expression of genes related to cell proliferation, DNA synthesis, and cytoskeletal remodeling [138] (Figure 2d). Furthermore, YAP1 stimulates IGF-1 and Akt signaling to reduce cardiomyocyte apoptosis [44]. Hence, in response to heart injury, YAP1 could stimulate the onset of all phenotypes that allow cardiomyocytes to enter a cardiac regeneration program.

Heallen and coworkers demonstrated how the shutdown of the Hippo cascade (by either in vivo SAV1 inactivation or LATS1/2 inhibition) in postnatal cardiomyocytes promoted efficient heart regeneration after cardiac apex resection and subsequent myocardial infarction (MI) in terms of cell proliferation and functional heart recovery [139]. Likewise, in mice with established ischemic HF, cardiomyocyte-specific deletion of SAV1 3 weeks after MI resulted in increased cardiomyocyte proliferation, scar size reduction, and enhanced heart function [140]. Moreover, it was reported that adeno-associated virus 9 (AAV9)-YAP1 delivery (the human constitutive active form YAP1^S127A^) in mouse cardiomyocytes promoted cardiomyocyte division by boosting the cell cycle, as detected by the 4/5-fold higher presence of pHH3 and 5-ethynyl-2 deoxyuridine (EdU) uptake; this was sufficient to reduce scar size and to increase cardiac function in terms of ejection fraction and the attenuation of cardiac remodeling (hypertrophy) after MI, without signs of apoptosis [106].

YAP1, once in the nucleus, triggers the transcription of a series of prosurvival genes that compete for cardiac regeneration. Additionally, it can interact with other molecular partners to accomplish its fate. For example, Tao G. and colleagues found a functional interaction between YAP1 and paired-like homeodomain transcription factor (PITX2) in injured SAV1-deficient hearts [141]. PITX2 mitigates detrimental ROS effects by modulating the main scavenger enzymes and improving the function of ETC components. In support of this finding, conditional KO of PITX2 in neonatal mouse hearts resulted in an impaired regenerative response after apex resection, while PITX2 gain of function in adult cardiomyocytes allowed cardiac repair and function [141].

In addition to genetic animal models showing regenerative properties after the forced induction of YAP1 translocation into the nucleus, upstream effectors modulating its physiological translocation also exist. Interestingly, Bassat and coworkers have proven that Agrin (Agrn), a protein of the extracellular matrix, is one of them. Indeed, its conditional KO in mouse heart impaired cardiac regeneration after apex resection, as observed by increased fibrosis and reduced cardiomyocyte proliferation [142]. Moreover, they have also reported that Agrn interacts with its receptor a-Dystroglycan (Dag1) and promotes disassembly of the dystrophin–glycoprotein complex (DGC). This event triggers YAP1 translocation to the nucleus, where it promotes cardiomyocyte proliferation [142]. In accordance with these findings, Morikawa et al., in 2017, also observed that functional DGC interacts with YAP1 to prevent its nuclear translocation and inhibits cardiomyocyte proliferation [143].

Although the inhibition of Hippo kinases and YAP1 activation have been proven to stimulate myocardial regeneration after cardiac injury, the long-term consequences of chronic Hippo pathway shutdown remain to be seen. To the best of our knowledge, long-term expression (some months) of YAP1 in the heart is accompanied by a slight increase in fibrosis, no signs of hypertrophy, and no tumor-associated manifestations, but a mild decrease in cardiac function. It should be seen if the longest periods are well tolerated.

## 5. Contribution of the Hippo Pathway in Inflammatory States

As inflammation is one of the pathways that significantly drives cardiac diseases together with mitochondrial dysfunctions and ROS production, it deserves a brief summary from the Hippo pathway point of view. Indeed, intensive research in the last few years has revealed that Hippo signaling is also involved in the occurrence and progression of inflammation [144]. Inflammation is a complicated process composed of different mechanisms [145]; basically, it constitutes a fundamental protective response, but it could be one of the primary contributors to the pathogenesis of several chronic diseases.

It was pointed out that in *Drosophila*, Gram-positive bacteria act as extracellular stimuli of Hippo signaling under physiological settings, leading to a decrease in antimicrobial peptide secretion and restriction of inflammation [146]. YAP1 and TAZ were reported to be mediators of many inflammatory processes [147,148]. Moreover, both NDR1/2 and MST1/2 have also been implicated in inflammation in recent years [149]. The kinase module influences different important determinants of the immune response, such as T-cell survival, adhesion, chemotaxis, and proliferation [150]. Apart from directing the innate immune response, the Hippo pathway components also modulate the adaptive immune responses in multiple pathological conditions. For example, it has been shown that YAP1/TAZ expression in the epicardium is essential not only for coronary vasculature development [151] but also for limiting the inflammatory and fibrotic response during the post-MI recovery phase through recruitment of Tregs [152]. Furthermore, Ramjee et al. demonstrated that the loss of YAP1/TAZ in the epicardium resulted in decreased expression of IFN-γ, a known Treg inducer [153]. After MI stress, a rapid inflammatory response is activated in the myocardium. This process is necessary to clear debris and promote wound healing after injury. Nevertheless, excessive inflammation can increase matrix degradation, cause greater cardiomyocyte loss, augment fibrosis, and worsen heart function. Therefore, a balanced response is fundamental to providing optimal cardio protection. The role of the Hippo signaling components in nonimmune cells regulating the inflammatory response is well established; however, a growing literature is emerging about its functions in immune cells. Recent studies have revealed that YAP1 is an unexpected amplifier of a Treg-reinforcing pathway [154], and in macrophages, it aggravates inflammatory bowel disease, accompanied by the production of antimicrobial peptides and changes in gut microbiota [155].

Recent works have demonstrated that TAZ regulates T-cell differentiation in both mouse and human memory CD4^+^ T cells. TAZ promotes TH17 cell development, a proinflammatory subtype that is involved in autoimmunity while attenuating Treg cell production [156]. TAZ is directly bound to RAR-related orphan receptor C to promote the TH17 subset and potentiated autoimmune disease, defining a role as a negative regulator of adaptive immune responses for the Hippo pathway.

In a recent study, Mia et al. demonstrated that in cardiac fibroblasts, YAP1/TAZ are essential regulators of macrophage polarization and functions by regulating interleukin 6 (IL6) promoter activity or through the p38-dependent MAPK pathway after MI. In response to both proinflammatory and reparative stimuli, YAP1 and TAZ expression is increased in macrophages. Interestingly, their data demonstrate that YAP1/TAZ act as activators in proinflammatory macrophages (M1 phenotype) while behaving as repressors in reparative macrophages (M2 phenotype) [157]. Importantly, Hippo signaling impacts adaptive and innate immune cell functions, modulating both pathogen-triggered responses, such as myocarditis and sterile inflammation, resulting from injury, such as MI.

## 6. Conclusions

In some fields of research (i.e., cancer and related inflammatory states), all of the intricacies of the Hippo pathway have been revealed and its targeting is already a reality in many of the current anticancer therapies, but the knowledge in the cardiovascular field is still a mystery with interesting (sometimes controversial) facts that should be better interconnected. Many findings concerning its relevance in physiology have been deeply investigated in *Drosophila* and other similar organisms, but the findings need to be translated to and confirmed in mammals and humans. The existence of several animal models with conditional activation/repression of each component of the Hippo cascade is crucial to further investigate their role in disease and explore possible therapies.

## Data Availability

Not applicable.
